# Effective Antifogging Coating from Hydrophilic/Hydrophobic Polymer Heteronetwork

**DOI:** 10.1002/advs.202200072

**Published:** 2022-03-14

**Authors:** Junhe Shi, Liju Xu, Dong Qiu

**Affiliations:** ^1^ Beijing National Laboratory for Molecular Sciences CAS Research/Education Center for Excellence in Molecular Sciences Institute of Chemistry Chinese Academy of Sciences Beijing 100190 China; ^2^ University of Chinese Academy of Sciences Beijing 100049 China

**Keywords:** adhesive, antifogging, coating, hydrophilic/hydrophobic heteronetwork

## Abstract

Fogging on optical devices may severely impair vision, resulting in unacceptable adverse consequences. Hydrophilic coatings can prevent surface fogging by instantly facilitating pseudo‐film water condensation but suffer from short antifogging duration due to water film thickening with further condensation. Here, an innovative strategy is reported to achieve longer antifogging duration via thickening the robust bonded hydrophilic/hydrophobic polymer heteronetwork coating to enhance its water absorption capacity. The combination of strong interfacial adhesion and hydrophilic/hydrophobic heteronetwork structure is key to this approach, which avoids interfacial failure and swelling‐induced wrinkles under typical fogging conditions. The developed antifogging coating exhibits prolonged antifogging durations over a wide temperature range for repetitious usages. Eyeglasses coated with this coating successfully maintained fog‐free vision in two typical scenarios. Besides, the coating recipes developed in this study also have potential as underwater glues as they demonstrate strong adhesions to both glass and polymer substrates in wet conditions.

## Graphical abstract

Prompt antifogging performance is achieved by a specially designed hydrophilic/hydrophobic polymer heteronetwork coating. The heteronetwork structure ensures strong and durable adhesion on two most used optical materials, glass, and poly(methyl methacrylate) (PMMA), therefore is readily applicable for antifogging spectacles. This work also sheds light on the design of universally strong adhesives for a range of substrates, both in air and under water.

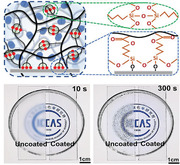

## 1 Introduction

Optical devices are prone to fogging (formation of tiny water droplets that distract the optical transmittance) when their surface temperature is approaching or below the dew point of the surrounding atmosphere, leading to substantially performance detriments or even disastrous consequences in health and safety.^[^ ^1^, ^2^, ^3^, ^4^, ^5^ ^]^ For instances, fog formed on the camera lens makes images blurred and distorted;^[^ ^6^ ^]^ drivers’ vision may be impaired by fog on the vehicle windscreen or rearview mirrors, which is one of the main causes for traffic accidents.^[^ ^7^ ^]^ In addition, fog on optical sensors or instruments often reduces the precision of spectrographs.^[^ ^8^ ^]^ Therefore, effective antifogging strategies for optical devices are highly demanded.

Antifogging coating is regarded as the most promising approach to avoid fog formation on optical devices, whereas the antifogging coating is mainly divided into two classes in terms of wettability: hydrophobic and hydrophilic. The hydrophobic coating works by reducing adhesion and enhancing repellence of water droplets to substrates.^[^ ^9^, ^10^, ^11^, ^12^ ^]^ As water droplets are consistently removed by gravity from the surface, hydrophobic coating is advantageous in long‐term effectiveness. However, water droplets can only be removed once they have grown above a critical size, i.e., 10 µm,^[^ ^13^, ^14^ ^]^ therefore, an induction antifogging period is inevitable for hydrophobic coating, which, although may be very short, is still unacceptable for many circumstances. In the case of hydrophilic antifogging coating, condensed water spreads into a pseudo‐film shape immediately to avoid fogging,^[^ ^15^, ^16^, ^17^, ^18^ ^]^ thus it does not have the problem of induction antifogging period as the hydrophobic coating does. However, once the water film on surface grows to a certain thickness, it becomes mobile, which often results in image distortion.^[^ ^19^ ^]^ Therefore, hydrophilic antifogging coating suffers from short‐term effectiveness.

A methodology to avoid forming thick water film on hydrophilic coating will make this antifogging method both prompt and durable, till the temperature difference between surface and vapor becomes so small that further condensation is suppressed. Hydrophilic coating of polymer network can absorb water through volume swollen, therefore, increasing coating thickness may enhance its water absorption capability and prolong the antifogging duration.^[^ ^19^, ^20^, ^21^ ^]^ However, thick hydrophilic polymer coating raises the following primary challenges:^[^ ^22^, ^23^, ^24^, ^25^ ^]^ (i) maintaining high optical transmittance; (ii) avoiding wrinkles and creases in repeated drying‐swelling cycles; (iii) preventing peeling and cracking under fogging conditions. The principles and procedures for adhesion and microstructure regulation of hydrophilic polymer networks will have to be addressed before they can be used as effective antifogging coatings in practice.

Herein, we achieve long‐term antifogging performance on transparent substrates made of both silicate glass and polymethyl methacrylate (PMMA), by strongly bonded coating of a hydrophilic/hydrophobic polymer heteronetwork composed of hydrophilic poly(vinyl alcohol) (PVA) and hydrophobic poly 3‐(trimethoxysilyl) propyl methacrylate (PTPM), denoted as PVA/PTPM HN thereafter. Concretely, the antifogging duration is remarkably extended through increasing the thickness of the PVA/PTPM HN coating to enhance its water absorption capacity. To provide robust adhesion, the PVA/PTPM HN is coupled with the surfaces through either covalent bonding^[^ ^26^ ^]^ or topological entanglements.^[^ ^27^, ^28^ ^]^ Meanwhile, the hydrophobic PTPM in heteronetwork restrains the over‐swelling of hydrophilic PVA network,^[^ ^29^, ^30^ ^]^ thus preventing wrinkle formation. The resultant coated slides can effectively avoid fog formation and maintain high transmittances (>85%) in high humidity environments over a wide temperature range (20–100 ℃) for as long as 30 min, one of the best among its peers.^[^ ^31^, ^32^, ^33^, ^34^ ^]^ Furthermore, we demonstrate the excellent antifogging effect of PVA/PTPM HN coating on eyeglasses, paving the way for such a technology to commercial applications. As the lack of adhesion and over‐swelling are two well‐known deficiencies of hydrogels,^[^ ^35^, ^36^ ^]^ the progress made in this study also enables hydrogels to be used in many other fields,^[^ ^37^, ^38^, ^39^, ^40^ ^]^ for example, under water adhesives.

## 2 Results and Discussion

### 2.1 Design of Thick Hydrophilic/Hydrophobic Polymer Heteronetwork Coating for Effective Antifogging Performance

We proposed a general strategy to achieve long‐term antifogging performance via thickening the robust bonded hydrophilic/hydrophobic polymer heteronetwork coatings. The hydrophilic polymer coatings are well known to have antifogging performance by swiftly spreading and absorbing condensed water.^[^ ^8^, ^41^ ^]^ However, these hydrophilic coatings can only immobilize limited amount of water, and a mobile water film is quickly formed afterward. Increasing the thickness of hydrophilic coatings may delay the formation of mobile water film to a time point, at which the surface is sufficiently warmed up and no more condensed water is generated, thus to achieve effective long‐term antifogging performance. Nevertheless, the issues arisen with thicker coating have to be addressed before hand.

**Figure 1:**
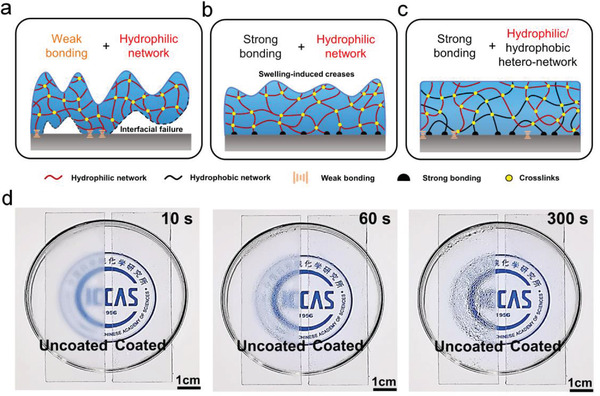
Design of the thick hydrophilic/hydrophobic polymer heteronetwork coating for effective antifogging performance. a) Weak adhesion of hydrophilic polymer network coating results in interfacial failure along with breakages and wrinkles with increasing thickness. b) Swelling induces creases on the surface of strongly bonded hydrophilic polymer network coating with increasing thickness. c) Robust anchorage of hydrophilic/hydrophobic polymer heteronetwork coating avoids both interfacial failure and creases at larger thickness. The antifogging duration is prolonged by higher water absorption in the thick hydrophilic/hydrophobic polymer heteronetwork coating. d) Antifogging behavior of a pristine glass slide (uncoated) and the one coated with PVA/PTPM HN (coated) in hot water vapor (60 ℃) for 10, 60, and 300 s. The coated glass slide remained clear until 300 s, while the uncoated one became blurred even at 10 s.

Weak bonding of coating on surface often leads to interfacial failure along with breakages and wrinkles at increased thickness (**Figure** **1**a). Although increasing bonding strength can effectively avoid interfacial failure, the inevitable inhomogeneity in polymer network is amplified during the swelling of thicker network, i.e., forming wrinkles or creases (Figure 1b),^[^ ^42^, ^43^ ^]^ which either blur the vision or distort the image. We therefore propose to use thick hydrophilic/hydrophobic polymer heteronetwork coating with strong interfacial bonding for persistent antifogging performance. The hydrophobic moiety in the heteronetwork inhibits the formation of large‐scale inhomogeneity by preventing any local over‐swelling of hydrophilic polymer segments, thus, to maintain long‐term antifogging performance (Figure 1c). The obtained glass slide coated with a 100 µm thick PVA/PTPM HN showed persistent antifogging ability, remaining clear for at least 300 s over a 60 ℃ water bath, while the uncoated one became blurred even at 10 s (Figure 1d).

### 2.2 Construction of robust PVA/PTPM HN Antifogging Coating

**Figure 2:**
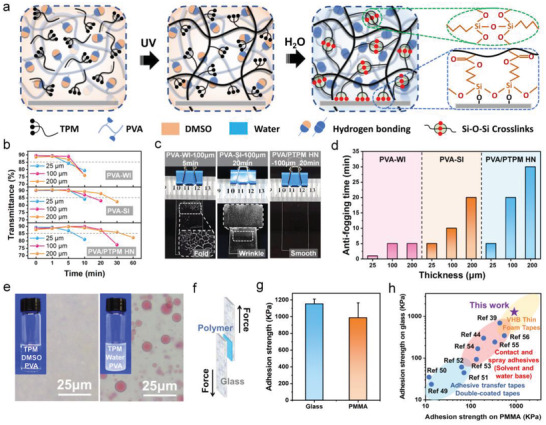
Antifogging performance of PVA/PTPM HN coating and the mechanism of its long‐term effectiveness. a) Illustration of coating PVA/PTPM HN on silicate glass via a two‐step method involving photopolymerization and solvent exchange. b) The average transmittance of PVA‐WI, PVA‐SI, and PVA/PTPM HN coated glass slides with varying coating thickness over time when exposed to hot water vapor (60 ℃). c) Optical photographs of the surface of 100 µm thick PVA‐WI, PVA‐SI, and PVA/PTPM HN coatings after exposed to hot vapor (60 ℃) for 5 (left), 20 (middle), and 20 min (right), respectively. d) Antifogging duration of PVA‐WI, PVA‐SI, and PVA/PTPM HN coatings with varying thickness. e) The optical microscope images of the mixtures of TPM/PVA in DMSO (DMSO route) and TPM/PVA in water (water route) dyed with Sudan III, showing the homogeneous dissolution of TPM in DMSO while phase separation of TPM in water. The insets are their corresponding optical images. f) Schematic illustration of measurement of adhesive strength based on the lap‐shear test. g) Wet‐contact adhesion strength of PVA/PTPM HN on glass and PMMA substrates. h) Comparison of adhesion strengths of the wet adhesive PVA/PTPM HN on glass and PMMA substrates to other polymer adhesives reported in the literature (point) and commercial 3M adhesives (colored area). The error bars represent standard deviation; sample size *n* = 3.

Our method to construct the hydrophilic/hydrophobic polymer heteronetwork antifogging coating is based on a two‐step approach involving the sequential processes of photopolymerization and solvent exchange (**Figure** **2**a). We first dissolved the hydrophilic PVA and hydrophobic 3‐(Trimethoxysilyl) propyl methacrylate (TPM) monomer in a cosolvent dimethyl sulfoxide (DMSO). PVA was chosen as the hydrophilic moiety because it has abundant hydrophilic hydroxyl groups to facilitate the quick spreading and sucking of condensed water (Figure S1, Supporting Information). TPM was photopolymerized in situ to form hydrophobic PTPM initiated by 365 nm ultraviolet, confirmed by the disappearance of C═C double bond (1635 cm^−1^) in Fourier transform infrared spectroscopy (FTIR, Figure S2, Supporting Information) and the increase in viscosity (Figure S3, Supporting Information). The mass ratio of PVA/PTPM in the antifogging coating was set as 7/1 to achieve both high transmittance and strong adhesion at increased thickness (Figure S4, Supporting Information). Upon displacing DMSO with water, interpolymer hydrogen bonding was restored to form hydrophilic PVA network.^[^ ^44^ ^]^ At the same time, the siloxane moieties on PTPM hydrolyzed then condensed to form hydrophobic PTPM network interpenetrating with the hydrophilic PVA network. Notably, this hydrophobic PTPM network offers dual functions: providing strong interfacial bonding by forming siloxane bonds with glass^[^ ^26^, ^45^ ^]^ and preventing the local over‐swelling of hydrophilic PVA network,^[^ ^46^, ^47^, ^48^ ^]^ thus avoiding interfacial failure and creases of coating.

### 2.3 Antifogging Performance of PVA/PTPM HN Coating

As a proof‐of‐concept, PVA/PTPM HN at various thickness (25, 100, and 200 µm) were coated on glasses using the procedures proposed in Figure 2a. Other two control c were used for comparison: the PVA coating by drying aqueous PVA solution on the glass slide at 25 ℃ for 24 h, representing the hydrophilic coating with weak interfacial bonding (named as PVA‐WI); and covalently anchored PVA coating on the glass slide pre‐modified with epoxide groups, under otherwise identical conditions, representing the hydrophilic coating with strong interfacial bonding (named as PVA‐SI, Figure S5, Supporting Information). The average transmittance between 400 and 800 nm wavelength (obtained by Equation S1, Supporting Information) is used to assess their antifogging performance (Figure S6, Supporting Information).

As shown in Figure 2b and Figure S7 (Supporting Information), despite of the increase in PVA‐WI coating thickness from 25 to 200 µm, sharp reduction of optical transmittance was still observed after only 5 min, accompanied with interfacial breakages and wrinkles in the coating (Figure 2c, left). With the enhancement of interfacial bonding (PVA‐SI coating), although coating breakages were avoided, surface creases were still evident (Figure 2c, middle), leading to the decrease in light transmittance at only slightly prolonged durations (Figure 2b). In contrast, with the aid of hydrophobic moieties (PVA/PTPM HN), neither interfacial breakages nor creases were observed under otherwise the same conditions (Figure 2c, right), consequently, the decrease in average transmittance was remarkably delayed upon increasing the thickness (Figure 2b), maintaining a clear vision for as long as 20 min. Herein, we take an average transmittance higher than 85% as a successful antifogging effect. As summarized in Figure 2d, the antifogging duration of PVA/PTPM HN coatings increased remarkably, from 5 to 30 min when increasing their thickness from 25 to 200 µm, which was attributed to the enhanced water absorption capacity (Figure S8, Supporting Information), while that of other two control samples, PVA‐WI and PVA‐SI coatings, showed no obvious enhancement, manifesting the rationality of our strategy for antifogging coating design. This prolonged antifogging duration may enable the warm‐up of substrates by environment, thus eventually avoid fog formation in practice.

### 2.4 Structural and Mechanical Characteristics of PVA/PTPM HN Antifogging Coating

The role of DMSO is nontrivial; it enables a well mixing of PVA and TPM, therefore guarantees the formation of a homogeneous and interlaced hydrophilic/hydrophobic polymer heteronetwork structure, essential for good optical transparency (Figure 2e; Figure S9, Supporting Information). By contrast, phase separation of TPM was observed in water and an opaque gel was obtained after polymerization of TPM through water route (Figure S10, Supporting Information). Consequently, the PVA/PTPM (DMSO route) coated glass slides remained transparent at 200 µm thickness (transmittance higher than 85%), while the PVA/PTPM (water route) coated ones became blurred even at 25 µm thickness (transmittance less than 85%), as shown in Figure S11 in the Supporting Information.

PTPM forms covalent bonding with glass surface through the condensation with silanol groups, which remarkably increases the adhesive strength of PVA/PTPM HN coatings. The lap‐shear test (Figure 2f) revealed a rather strong adhesion of two glass slides glued by PVA/PTPM HN coating, i.e., ≈1171 ± 147 KPa at dry state and ≈1153 ± 58 KPa at wet state (Figure 2g; Figure S12, Supporting Information), enabling a lift of a 5 kg hydrothermal reactor both in air and water with an overlap area of 25 mm × 25 mm (Figure S13, Supporting Information). Microscopically, the PVA/PTPM HN coating exhibited a mixed failure mode on glass slides (Figure S14, Supporting Information), manifesting its robust adhesive feature. In addition, the measured interfacial toughness of the developed PVA/PTPM HN coating on glass substrate by peeling test is over 350 J m^−2^ (Figure S15, Supporting Information), also manifesting its robust adhesive ability. On the other hand, for polymeric substrates without surface hydroxyl groups, strong bonding of PVA/PTPM HN coating can also be realized via topological entanglements, benefited from the good swelling capability of DMSO to most polymers, again highlighting the advantage of DMSO route. Taking the most common optical polymer, PMMA, as an example, this topological entanglement is achieved by partial swelling of surface with TPM/DMSO solution followed by subsequent photopolymerization (Figure S16, Supporting Information). As expected, strong adhesive strength (914 ± 80 KPa at dry state and ≈987 ± 178 KPa at wet state) on PMMA substrates was also obtained (Figure 2g; Figure S12, Supporting Information). On the contrary, in the absence of covalent bonds and/or topological entanglements, PVA adhesion strength was much lower, i.e., ≈359 ± 60 KPa for glass and ≈52 ± 16 KPa for PMMA (Figure S17, Supporting Information). Indeed, the wet‐contact adhesive strengths of the PVA/PTPM NH coatings on both silicate glass and PMMA were much higher than many commercial glues and high‐performance polymeric adhesives in literatures (Figure 2h).^[^ ^39^, ^44^, ^49^, ^50^, ^51^, ^52^, ^53^, ^54^, ^55^, ^56^, ^57^ ^]^ Even for two surfaces of dissimilar materials, this PVA/PTPM NH could still glue them. For examples, the wet‐contact adhesive strengths between glass slides to steel, aluminum and PMMA slides were found to be ≈1091 ± 159, ≈1082 ± 128, and ≈839 ± 83 KPa, respectively (Figure S18, Supporting Information). Therefore, this strategy developed for antifogging coating also sheds light on the design of universal strong adhesives for materials ranging from glass through metal to polymer, both in air and under water.

It is worth noting that PVA/PTPM HN coating on PMMA substrate also possessed an effective antifogging performance, with the antifogging duration of ≈30 min at a thickness of 200 µm (Figure S19, Supporting Information). On the contrary, the antifogging duration of PVA‐WI coatings on PMMA (prepared following the same procedure as PVA‐WI coating on glass slides) was only ≈10 min under otherwise the same conditions (Figure S19, Supporting Information). In addition, the PVA/PTPM HN coating still maintained high transparency (above 85%) both at dry and wet states even after a 5 N load of dynamic scratching, attributed to its high hardness (Figures S20 and S21, Supporting Information). Thus, the combination of homogeneous hydrophilic/hydrophobic heteronetwork structure and strong adhesion and scratching resistance in PVA/PTPM HN coatings endues them with the potential of universal antifogging coatings on diverse optical materials.

### 2.5 Temperature Tolerance and Reusability of the PVA/PTPM HN Antifogging Coating

**Figure 3:**
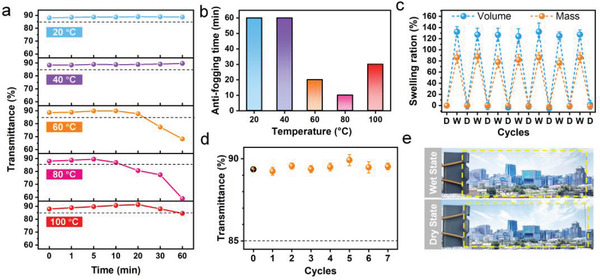
Temperature tolerance and reusability of the PVA/PTMP HN antifogging coating on glass slides. a) The average transmittance of the PVA/PTPM HN coated glass slides at different temperatures. b) Antifogging duration of PVA/PTPM HN coatings over a wide temperature range. c) The volume and mass swelling ratio of PVA/PTPM HN coating during cyclic drying‐swelling test, where D and W denote the dry and wet state, respectively. d) The average transmittance of PVA/PTPM HN coated glass with a coating thickness of 100 µm through repeated antifogging tests on a 60 ℃ water bath, indicating its excellent reusability. e) The optical photographs of dry‐state (top) and wet‐state (bottom) of PVA/PTPM HN coated glass slides after 7 cycles of antifogging tests, showing a clear vision over the same picture. The error bars represent standard deviation; sample size *n* = 3.

Water vapor temperature has a profound effect on antifogging performance due to the altered condensation speed and size of the condensed water droplets. To further investigate the temperature impact, optical transmittance of PVA/PTPM HN coated glass slides with the coating thickness of 100 µm was measured over time at different temperatures (Figure S22, Supporting Information). As shown in **Figure** **3**a, these coated glass slides maintained a high optical transmittance (over 85%) almost independent with time at temperatures below or at 40 ℃. At higher temperatures, reduction in optical transmittance was observed after an initial plateau and became lower than 85% at ≈20 and ≈10 min for 60 and 80 ℃, respectively, probably due to the formation of excess water layer beyond its water absorption limit.^[^ ^19^ ^]^ Surprisingly, the decrease in optical transmittance was suppressed at 100 ℃, probably due to the approaching of water‐vapor phase equilibrium point.^[^ ^58^ ^]^ Overall, as summarized in Figure 3b, the PVA/PTPM HN coating can effectively prevent fog formation on glass slides over a wide temperature range (20–100 ℃).

Reusability is a key challenge faced by existing antifogging approaches but essential for practical applications, as the coating will have to endure repeated swelling–drying cycles. We explored the reusability of PVA/PTPM HN coating through repeated antifogging tests. In the initial swelling process, the equilibrium mass and volume swelling ratios of PVA/PTPM HN coating can reach 86 ± 11% and 133 ± 17% within 30 min (Figure S23, Supporting Information). After seven cycles of drying‐swelling tests, they maintained fairly steady levels comparable to the original ones, i.e., ≈130% and ≈80%, respectively (Figure 3c), ensuring the long‐term and repetitious antifogging performance. In addition, the PVA/PTPM HN coating on glass slides demonstrated robust adhesion and remained smooth after immersed in water for up to 3 d (Figure S24, left, Supporting Information). In contrast, the PVA‐WI coating showed interfacial failure and wrinkled surface upon swelling in water, severely deteriorating the optical transparency (Figure S24, right, Supporting Information). Attributed to the strong interfacial adhesion and hydrophilic/hydrophobic heteronetwork structure, the PVA/PTPM HN coating on glass slides remained highly transparent (optical transmittance over 88%) during seven cycles of fogging tests (Figure 3d,e), manifesting its long‐term antifogging performance.

### 2.6 Pseudo‐Service Performance Evaluation of PVA/PTPM HN Antifogging Coating

**Figure 4:**
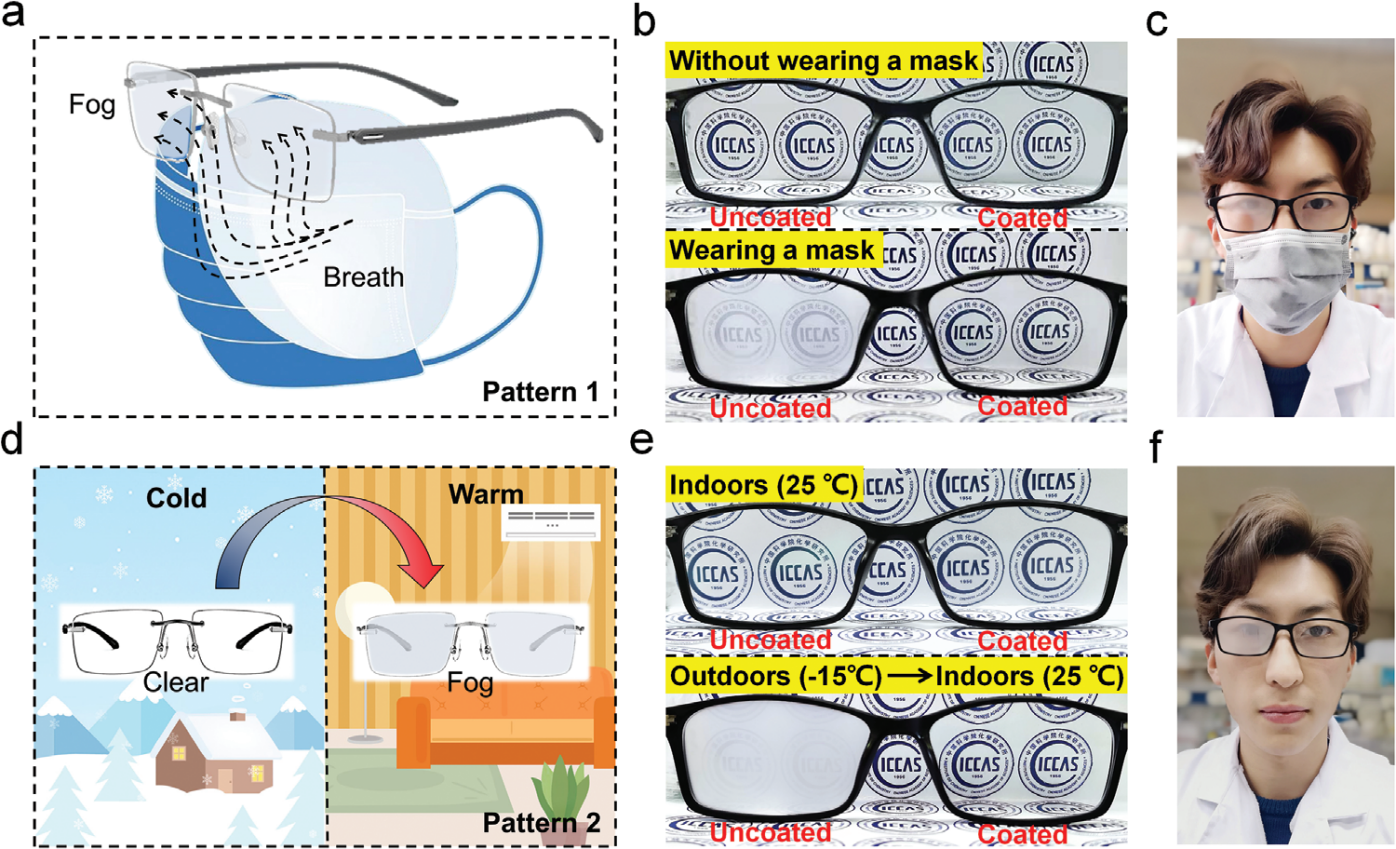
Antifogging eyeglasses using the PVA/PTPM HN coating. a) The schematic route of fogging on eyeglasses when wearing a mask. b,c) The antifogging test of eyeglasses when wearing a mask. d) The schematic illustration of fogging on eyeglasses when entering warm indoors from cold outdoors. e,f) The antifogging test of eyeglasses when entering indoors (25 ℃) from cold outdoors (−15 ℃).

Fog formed on optical devices, such as eyeglasses, may severely impair people's vision. Therefore, effective antifogging method is highly important. Owing to the outstanding antifogging performance of the PVA/PTPM HN coating, it can be readily used to endow eyeglasses with antifogging effect. The PVA/PTPM HN can be firmly coated on silicate‐based eyeglasses which are rich in surface silanol groups without sacrificing their inherent optical transparency. The antifogging performance of the coated eyeglasses was tested in two typical scenarios, where fog is prone to generate. In one case, a person simultaneously wore a pair of eyeglasses and a mask at room temperature (**Figure** **4**a–c). Fog easily formed on the uncoated lens (Figure 4b,c, left) due to the abundant water vapor from breathing, while the coated lens (Figure 4b,c, right) remained highly transparent. In the other case, a person wearing a pair of eyeglasses entered a warm room (25 ℃) from cold outdoors (−15 ℃) (Figure 4d–f). In this situation, the uncoated lens (Figure 4e,f, left) became opaque immediately. Notably, the coated one (Figure 4e,f, right) remained clear, manifesting the remarkable antifogging performance of PVA/PTPM HN coating. In addition, the coating is also much easier to clean once fouled by oily pollutants, such as annoying fingerprints (Figure S25, Supporting Information), which is highly valued for real‐world applications.

## 3 Conclusion

In summary, a highly adhesive hydrophilic/hydrophobic polymer heteronetwork thick coating is proposed to achieve effective antifogging performance. Under this concept, PVA/PTPM HN coatings are developed, starting from their solution in DMSO and finally crosslinked by photopolymerization and solvent exchange. These coatings displayed superior strong interfacial adhesion resulted from the formation of covalent bonds and/or topological entanglements with the targeted surfaces to avoid interfacial failure. Meanwhile, this hydrophilic/hydrophobic polymer heteronetwork restrains the formation of swelling‐induced wrinkles under typical fogging conditions. At increasing thickness, these coatings showed prolonged antifogging performance, when exposed to water vapor in a wide temperature range (20–100 ℃). Therefore, this PVA/PTPM HN antifogging coating offers advantages over existing antifogging materials, including longer antifogging duration, high transparency and stability over repeated usages. As a proof‐of‐concept, the PVA/PTPM HN coating demonstrated highly effective antifogging performance on the eyeglasses. We anticipate that the reported method and material are generally applicable for the rational design of high‐performance antifogging coatings towards commercial applications. Besides, the strategy developed for antifogging coating also sheds light on the design of universal strong adhesives for materials ranging from glass through metal to polymer, both in air and under water.

## 4 Experimental Section

### Preparation of PVA/PTPM HN Coating

The two‐step method involving photopolymerization and solvent exchange was adopted to prepare the PVA/PTPM HN coating. Typically, PVA with the mass of 3.6 g was dissolved in 15 mL DMSO and stirred for 2 h at 95 ℃. A mixture of TPM (0.5 mL) and DMPA (15 mg) was added to the above solution, followed by stirring for 20 min in dark. After defoaming, the resultant solution was spread onto the glass slides to produce a uniform liquid layer with different thickness (25, 100, 200 µm) using a wet film coater, followed by UV irradiation (365 nm wavelength, 0.8 W cm^−2^) for 1 h and solvent exchange in water for another 0.5 h. To obtained dry PVA/PTPM HN coated substrates, the samples were dried at 25 ℃ for 24 h.

### Statistical Analysis

When average value is referred, it is based on 3 parallel tests and presented in the form of mean ± SD. In the corresponding figures, error bars reflect the values of SDs.

## Conflict of Interest

The authors declare no conflict of interest.

## Supporting information

Supporting Information

Supplemental Movie 1

## Data Availability

The data that support the findings of this study are available from the corresponding author upon reasonable request.
