# Supplementary material for: Effective Antifogging Coating from Hydrophilic/Hydrophobic Polymer Heteronetwork
Source: Adv Sci (Weinh). 2022 Mar 14;9(14):2200072. doi: 10.1002/advs.202200072 (PMC9109053; doi:10.1002/advs.202200072)
Supplement: Supplementary file 1 — Supporting Information [file ADVS-9-2200072-s001.pdf]

ADVANCED SCIENCE
Open Access

Supporting Information

for *Adv. Sci.*, DOI 10.1002/advs.202200072

Effective Antifogging Coating from Hydrophilic/Hydrophobic Polymer Heteronetwork

*Junhe Shi, Liju Xu and Dong Qiu**

Supporting Information

Effective Anti-fogging Coating from Hydrophilic/Hydrophobic Polymer Hetero-Network

Junhe Shi[a,b], Liju Xu[a,], and Dong Qiu[a,b,*]*

a Beijing National Laboratory for Molecular Sciences, CAS Research/Education Center for Excellence in Molecular Sciences, Institute of Chemistry, Chinese Academy of Sciences, Beijing 100190, China

b University of Chinese Academy of Sciences, Beijing 100049, China

E-mail: xuliju@iccas.ac.cn; dqiu@iccas.ac.cn

Materials and methods

Materials. Polyvinyl alcohol 1799 (PVA-1799, 98-99% hydrolyzed, Aladdin), dimethyl sulfoxide (DMSO, Aladdin), 3-(Trimethoxysilyl) propyl methacrylate (TPM, Alfa), 2,2-bimethoxy-2-phenylacetophenone (DMPA, 98%, Alfa) and other chemicals (Sinopharm Chemical Reagent Co. Ltd.) were used without further purification. Pure water (18.2 MΩ·cm) was generated by an ELGA Purelab system.

Preparation of PVA-WI coating. PVA with the mass of 3.6 g was dissolved in 15 ml water and stirred for 2 h at 95℃. After defoaming, the resultant solution was spread on the glass slides to produce a uniform liquid layer with different thickness (25, 100, 200 μm) using a wet film coater. To obtained dry PVA-WI coated substrates, the samples were dried at 25℃ for 24 h.

Preparation of PVA-SI coating. The oxygen plasma-treated glass slides were first

immersed in a mixture solution with 90 ml ethanol, 10 ml water, 5 ml 3-glycidoxypropyltrimethoxysilane and 100 μl acetic acid at room temperature for 12 h. After thorough cleaning, the epoxy-functionalized glass slides were prepared. PVA with the mass of 3.6 g was dissolved in 15 ml water (pH = 2) and stirred for 2 h at 95℃. [1,2] After defoaming, the resultant solution was spread on the epoxy-functionalized glass slides to produce a uniform liquid layer with different thickness (25, 100, 200 μm) using a wet film coater. The samples were placed in 60℃ oven for 12 hours to complete chemical grafting of PVA. To obtained dry PVA-SI coated substrates, the samples were dried at 25℃ for 24 h.

Measurement of anti-fogging performance. The anti-fogging performance was characterized by a hot-vapor testing. Concretely, the samples were held above a water bath containing water with constant temperature, and the distance between the samples and the surface of the water was 5 cm. To investigate the anti-fogging properties of the samples, the light transmission over the 300-800 nm wavelength range was collected using a UV-Vis spectrophotometer during fogging tests. For comprehensive evaluation and direct comparison, the average transmission over the 400-800 nm wavelength was defined and obtained from the following equation:

$$T\% = \frac{\int_{400}^{800} x\, dx}{800 - 400}$$

The repeated anti-fogging tests were carried on a 60℃ water bath. The sample was first exposed to hot water vapor (60℃) for 10 min (denoted as the wet-state). After a drying process at 25℃ for 12 h (denoted as the dry-state), the second anti-fogging test

was carried, etc.

Measurement of volume and mass swelling ratio. The diameter, height and mass of the dry PVA/PTPM film was firstly measured as d_0, h_0 and m_0, and then immersed into water at 20℃. After reaching equilibrium swelling, the swollen PVA/PTPM film was taken out, and the diameter and height were measured again as d_w, h_w and m_w. The volume and mass swelling ratio denoted as Q_V and Q_M was obtained from the following equations:

$Q_V = (d_W*h_t - d_0*h_0)/d_0*h_0 \times 100\%$

$Q_M = (m_w - m_0) / m_0 \times 100\%$

Measurement of water contact angles. The water contact angles were measured using a contact angle meter (OCA25, Germany).

Attenuated total reflection Fourier transform infrared spectroscopy (ATR-FTIR). ATR-FTIR spectra were collected in wavenumber range of 4000-400 cm^{-1} on a Bruker Einox 55 instrument assisted by ATR attachments.

Lap shear adhesion test. The adhesive strengths of the PVA/PTPM and PVA network were investigated by shear-lap tests with two substrates of four materials (i.e., glass, PMMA, aluminum and steel). For adhesion tests, the adhesives (PVA/PTPM HN) were applied to one end of the adherend. This end was then covered by another adherend end with an overlap area of 25 mm × 25 mm in a lap shear configuration. The adherends were pressed together and allowed to keep in water for 5 days at room temperature before testing. Lap shear adhesion tests were performed on an Instron 3365 testing machine with a 5 kN load cell. The adherends were pulled apart at a

speed of 5 mm/min until failure occurred. The adhesion strength was calculated by dividing the force at failure with the overlap area.

90-dgree peeling test. The 90-dgree peeling test was performed with a universal tensile mechanical (Instron 5567) through pulling the gel sheet with a stiff backing from the substrate (i.e., glass). The peeling fixture maintained the peeling angle to be 90 degrees during the test via a pulley connected to the crosshead of the machine (test standard: ASTM D 2861). The rectangular hydrogels (80 mm× 20 mm× 2 mm) were used for the 90-dgree peeling test at a speed of 5 mm/min. The interfacial toughness was calculated by dividing the steady-state (or plateau) peeling force with the sample width.

Rheological measurements. The rheology experiment of the PVA-TPM and PVA/PTPM solutions (before and after UV irradiation) were measured at 25℃ using an TA Company's strain control rheometer ARES-G2. According to the concentration of solutions, the cone with the diameter of 25 mm and the cone angle of 1.024° was selected to test the rheological properties. The oscillation frequency sweep range was 0.1-100 Hz. Strain sweep was performed at a fixed frequency of 1 Hz for each sample, and the strain sweep range was 0.1 ~ 20% to determine the linear region. Finally, the strain of 5% was selected, which kept samples in the linear viscoelastic region.

Cryogenic scanning electron microscopy (Cryo-SEM) experiments. The specimens were transferred into a Cryo-preparation chamber (LEICA EM ACE600) under vacuum and sublimed by cooling to -100℃ for about 20 min. The frozen surface of the specimens was coated with Pt in order to make it conductive in an argon

environment (15 mA for 200 s). Then the specimens were transferred to the cryostage of -137℃ in the microscope (S-4300, HITACHI, Ltd, Japan). Finally, images were recorded using a 3 kV landing energy and 10 μA current.

Shore hardness test. The shore durometer was placed on the sample and tested by pressing.

Scratching test. The PVA/PTPM HN coatings at dry and wet states were repeatedly scratched using a piece of 500 g weight wrapped with a spectacle cloth. The transmittance of PVA/PTPM HN coatings was measured after 50 cycles of dynamic scratching.

Supplemental Movie

The movie showed the superb anti-fogging performance of the PVA/PTPM HN coated glass slide compared with the pristine one. When exposed to hot water vapor (60℃), the glass with PVA/PTPM HN coating maintained clear visions after 300 s, while the pristine glass slide showed blurred visions at 10 s.

Supplemental Figures

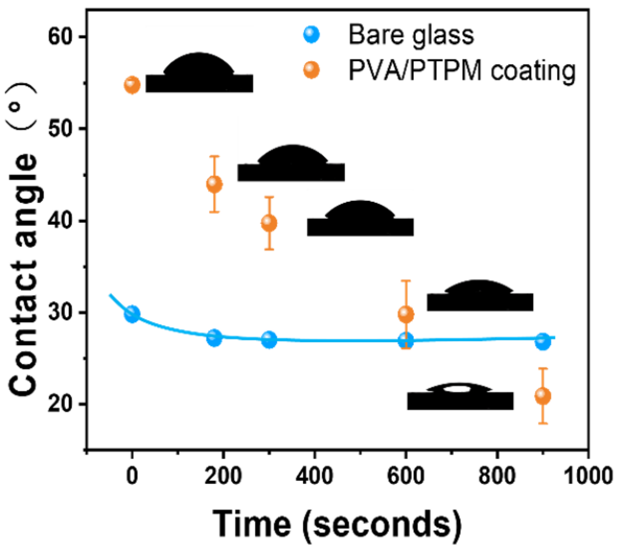

Figure S1. The water contact angle of the PVA/PTPM HN coating and bare glass over time.

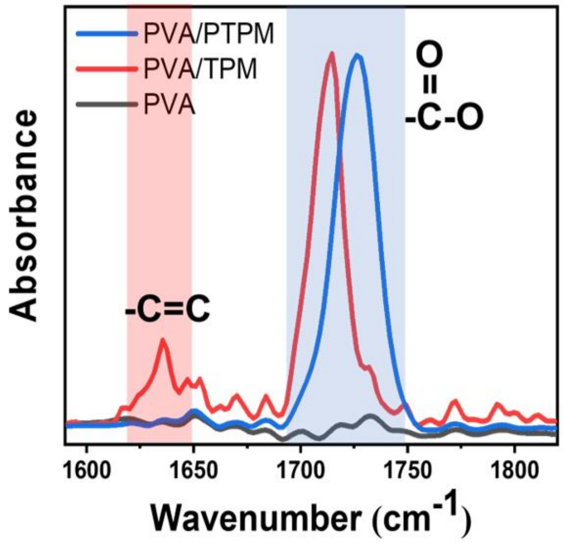

Figure S2. FT-IR spectra of the PVA/PTPM, PVA/TPM and pure PVA solutions. The absorption peak representing C=C double bond (1635 cm^{-1}) disappeared after photoinitiated polymerization. And the wavenumber of C=O-O ester group (1710 cm^{-1}) had a blue-shift to 1730 cm^{-1}, owing to the destroy of conjugated structure.

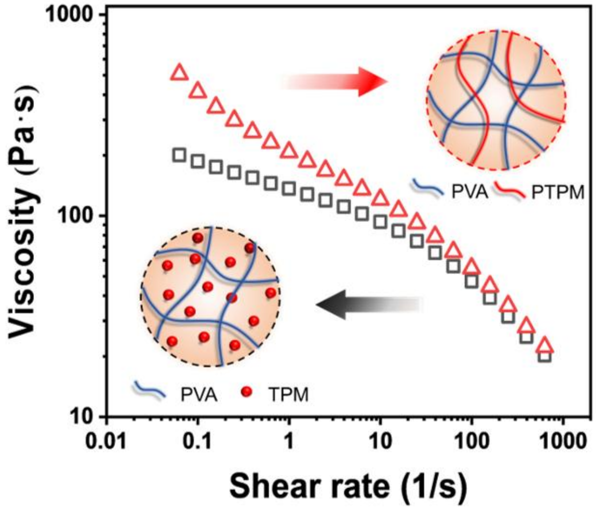

Figure S3 Rheological characterization of PVA/PTPM and PVA/TPM solutions. The

viscosity of the PVA/PTPM solution increased compared with the PVA/TPM solution, indicating the formation of PTPM network.

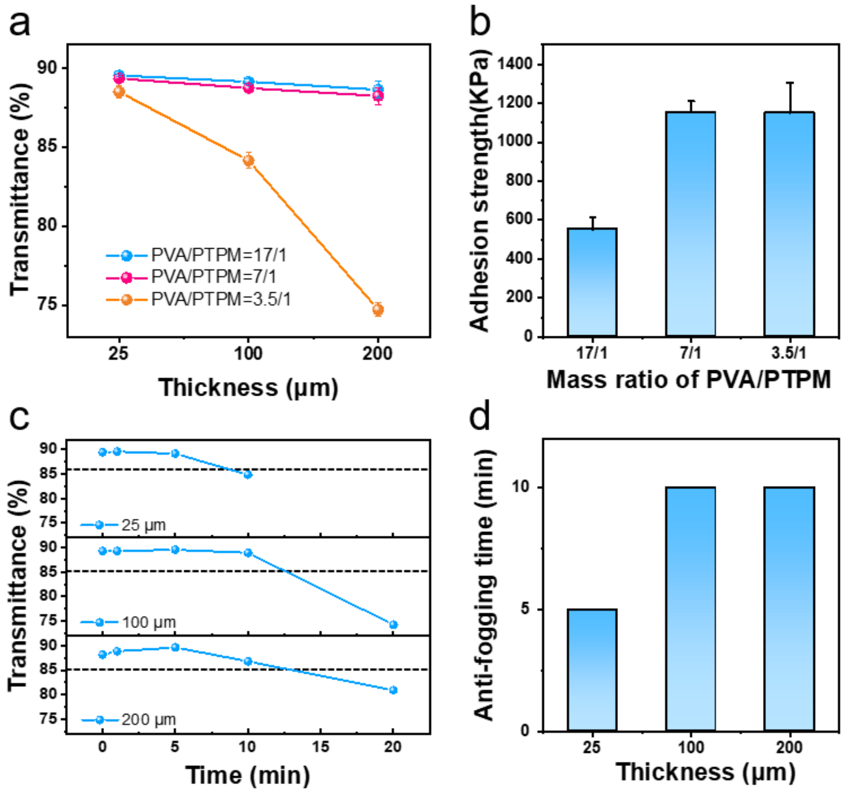

Figure S4. (a) The average transmittance of PVA/PTPM HN coatings with different mass ratio of PVA/PTPM and various thickness on glass slides. (b) Wet-contact adhesion strength of PVA/PTPM HN with different mass ratio of PVA/PTPM on glass. (c) The average transmittance of PVA/PTPM HN coatings with the PVA/PTPM mass ratio of 17/1 on glass with varying thickness over time when exposed to hot water vapor (60℃). (d) Anti-fogging duration of PVA/PTPM HN coatings with the PVA/PTPM mass ratio of 17/1 on glass with varying thickness.

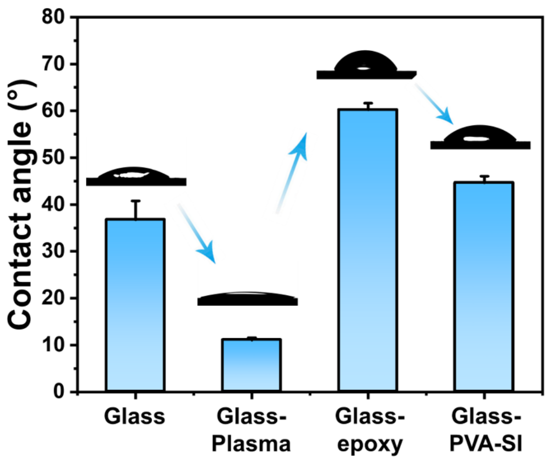

Figure S5. Contact angle measurements of the PVA-SI coating during the different preparation stage.

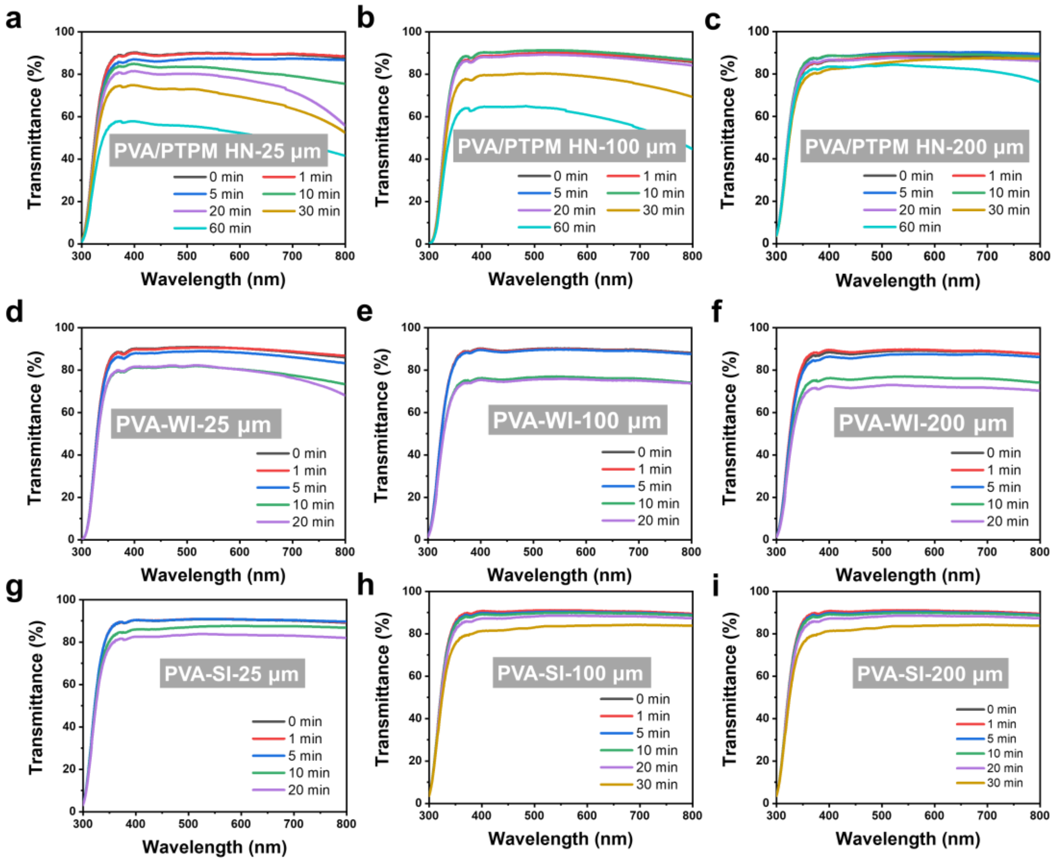

Figure S6. Transmission spectra of the (a-c) PVA/PTPM HN, (d-f) PVA-WI and (g-i) PVA-SI coatings on glass slides with the thickness of (a, d, g) 25 μm, (b, e, h) 100 μm

and (c, f, i) 200 μm.

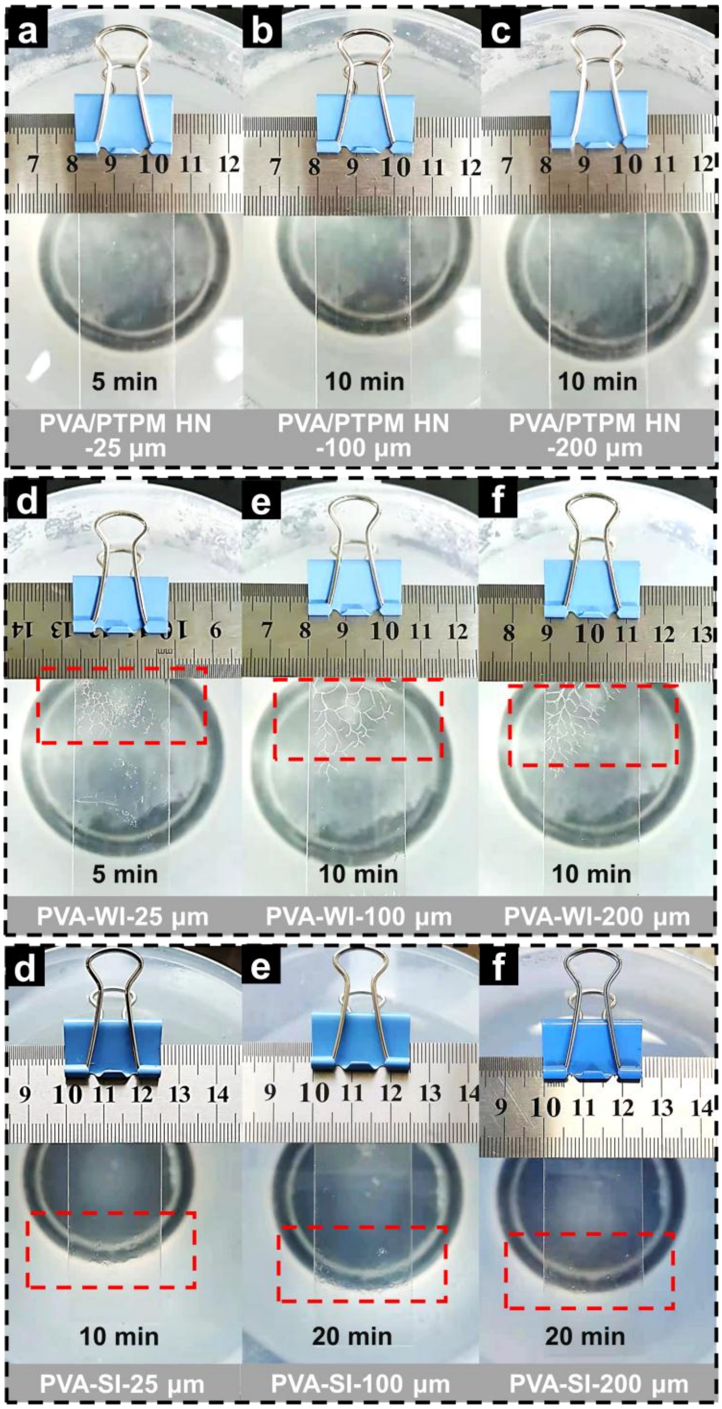

Figure S7. Surface morphology of (a-c) PVA/PTPM HN, (d-f) PVA-WI and (h-i) PVA-SI coatings on glass slides with the thickness of (a, d, g) 25 μm, (b, e, h) 100 μm, (c, f, i) 200 μm. The thickened fragile PVA-WI coating was prone to generate

wrinkles caused by the unstable adhesion and the PVA-SI tended to form image distortion as heterogeneous swelling behavior of PVA network, while the PVA/PTPM HN coating maintained smooth and transparency when simultaneously increasing the thickness and test time of anti-fogging, indicating the stable interfacial adhesion and uniform network structure.

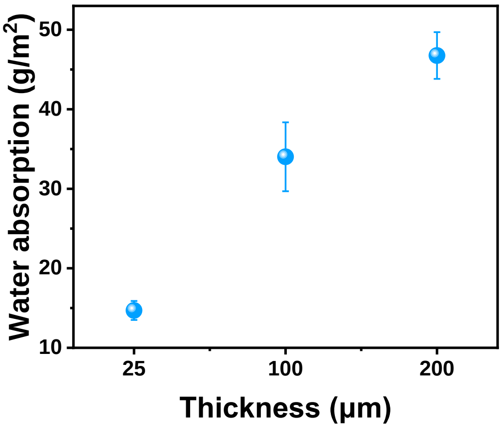

Figure S8. Water absorption of PVA/PTPM HN coatings with different thickness.

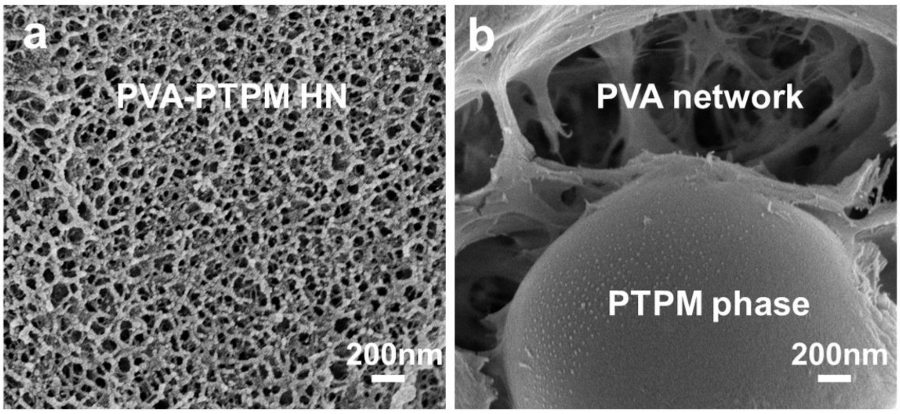

Figure S9. Cryo-SEM images of the (a) PVA/PTPM HN coating (DMSO route) prepared by solvent displacement from DMSO to water and (b) PVA/PTPM HN coating (water route) fabricated directly in water.

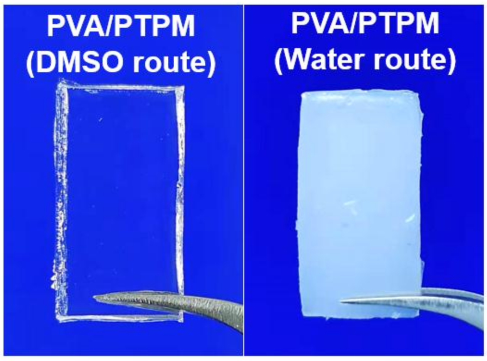

Figure S10. Optical photos of the PVA/PTPM gels prepared through DMSO route (left) and water route (right).

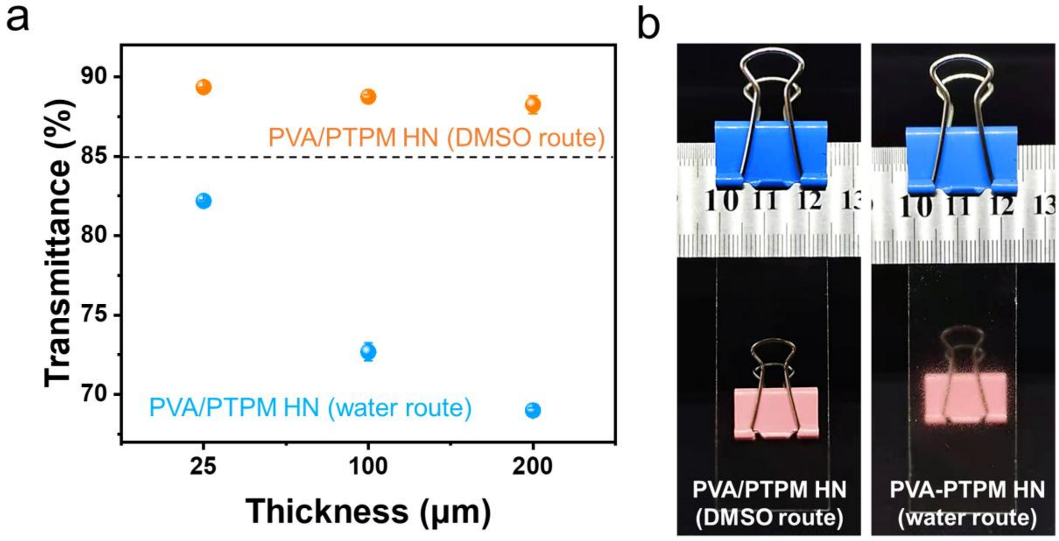

Figure S11. (a) The average transmittance of PVA/PTPM HN (Water route) and PVA/PTPM HN (DMSO route) coatings with various thickness on glass slides. (b) Optical images of PVA/PTPM HN (Water route) and PVA/PTPM HN (DMSO route) coatings with the same thickness of 100 μm on glass slides. Serious blur of image was observed on glass slide with PVA/PTPM HN (water route) coating caused by phase separation of TPM in water.

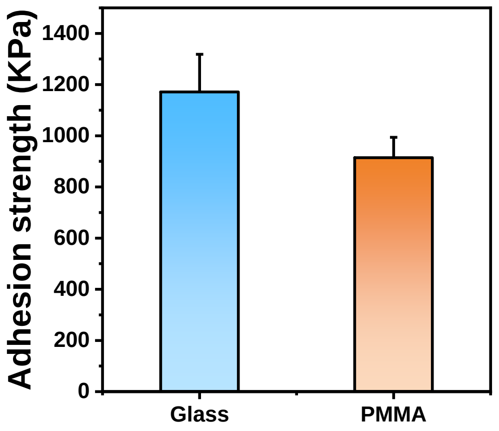

Figure S12. Adhesion strength of PVA/PTPM HN on glass and PMMA substrates at dry state.

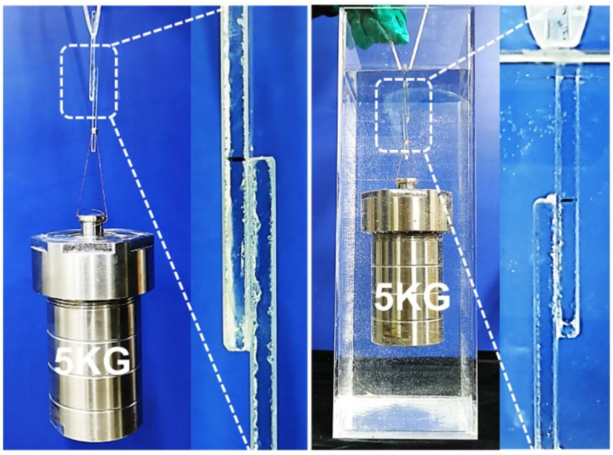

Figure S13. Optical images showing the bonded glass plates by PVA/PTPM HN can lift a hydrothermal reactor with a weight of 5 kg in air (left) and water (right).

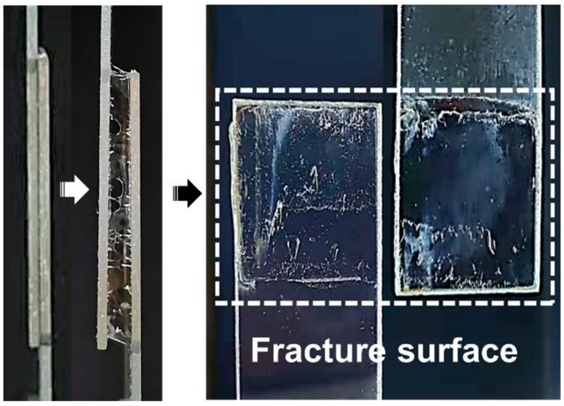

Figure S14. Optical images of mixed adhesive failure for two glass plates glued by the adhesive wet PVA/PTPM HN after a lap shear test.

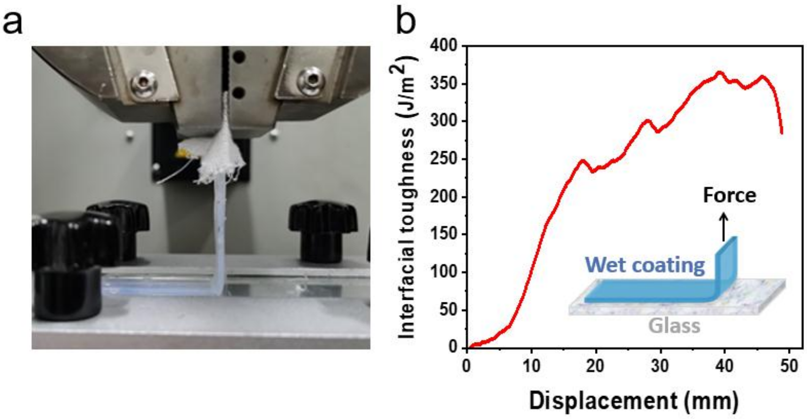

Figure S15. The 90-dgree peeling test of PVA/PTPM HN coating. (a) Photograph of PVA/PTPM HN coating during peeling test. (b) Interfacial toughness-displacement curves of PVA/PTPM HN coating.

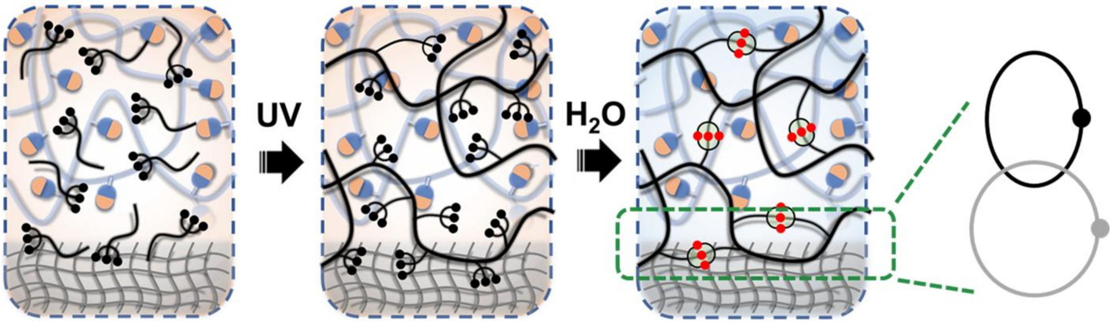

Figure S16. Mechanism for the strong adhesion of the wet PVA/PTPM HN on PMMA. The PMMA substrate can be swollen by DMSO and the TPM monomers can penetrate into the PMMA surface to form topological entanglements between PTPM and PMMA after polymerization, which contributed to the superb adhesion of the wet PVA/PTPM HN on PMMA.

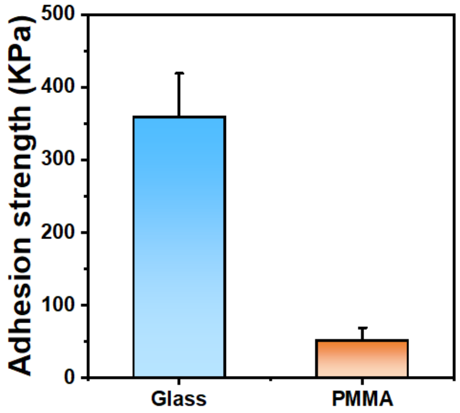

Figure S17. Wet-contact adhesion strength of the PVA network on glass and PMMA substrates.

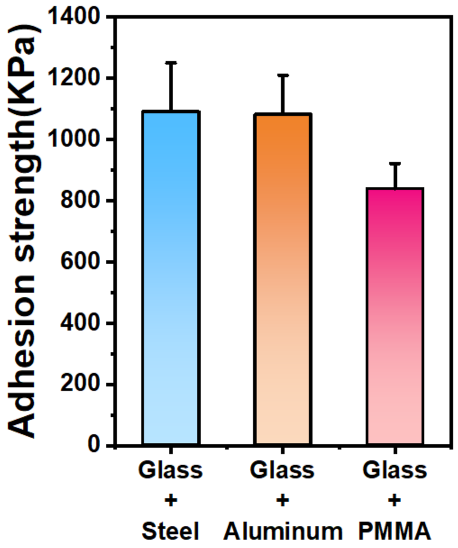

Figure S18. Wet-contact adhesion strength of PVA/PTPM HN gluing two dissimilar substrates with distinct properties.

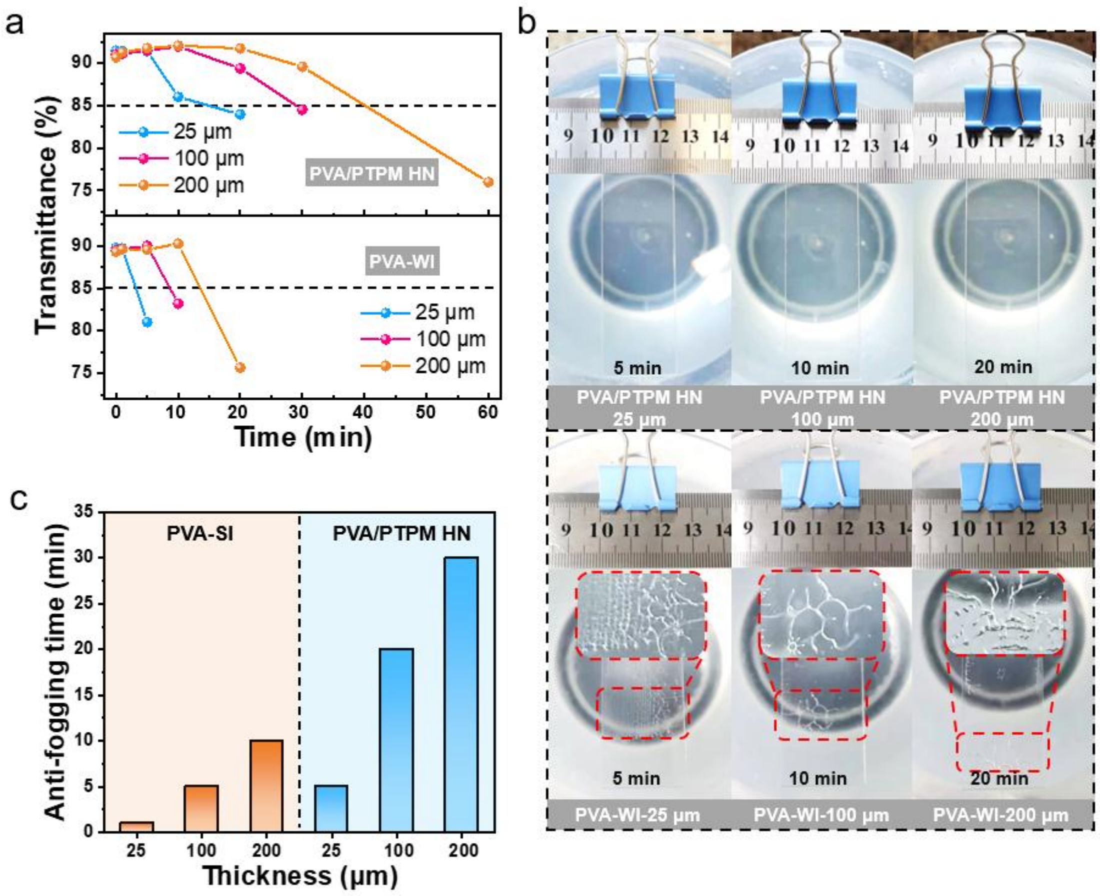

Figure S19. (a) The average transmittance of PVA-WI and PVA/PTPM HN coatings on PMMA with varying thickness over time when exposed to hot water vapor (60℃). (b) Surface morphology of PVA/WI (bottom) and PVA/PTPM HN (top) coatings on PMMA with the thickness of 25 μm, 100 μm, 200 μm after exposed to hot vapor (60℃) for 5 min (left), 10 min (middle) and 20 min (right), respectively. The thickened fragile PVA-WI coating was prone to generate wrinkles caused by the unstable adhesion, while the PVA/PTPM HN coating maintained smooth and transparency when simultaneously increasing the thickness and test time of anti-fogging, attributed to the stable interfacial adhesion and uniform network structure. (c) Anti-fogging duration of PVA-SI and PVA/PTPM HN coatings on glass slides with varying thickness.

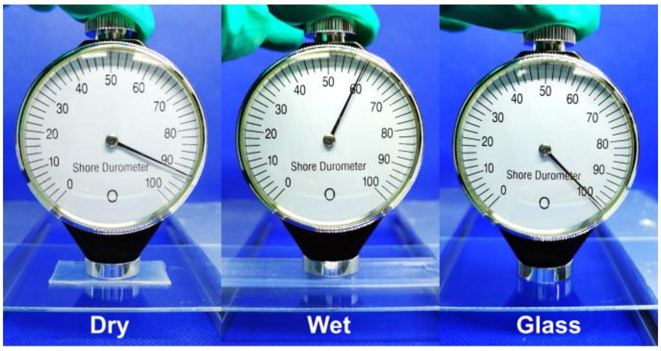

Figure S20. Shore hardness test of PVA/PTPM HN coating at dry and wet states.

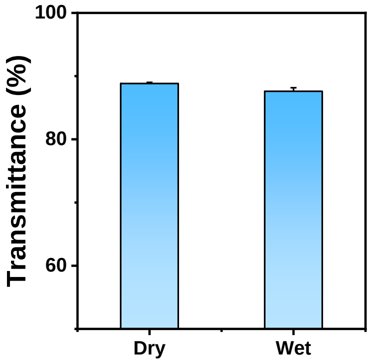

Figure S21. Measured transmittance of the PVA/PTPM HN coatings at dry and wet states after a 5 N load of dynamic scratching.

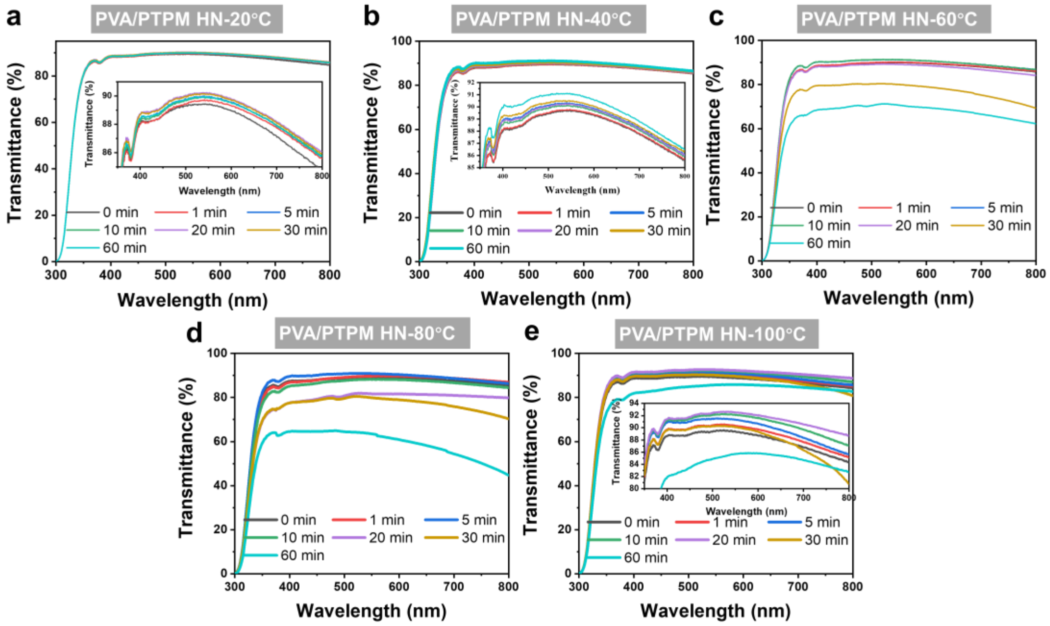

Figure S22. Transmission spectra of the PVA/PTPM HN coating on glass slides with the same thickness of 100 μm over time under (a) 20℃, (b) 40℃, (c) 60℃, (d) 80℃ and (e) 100℃.

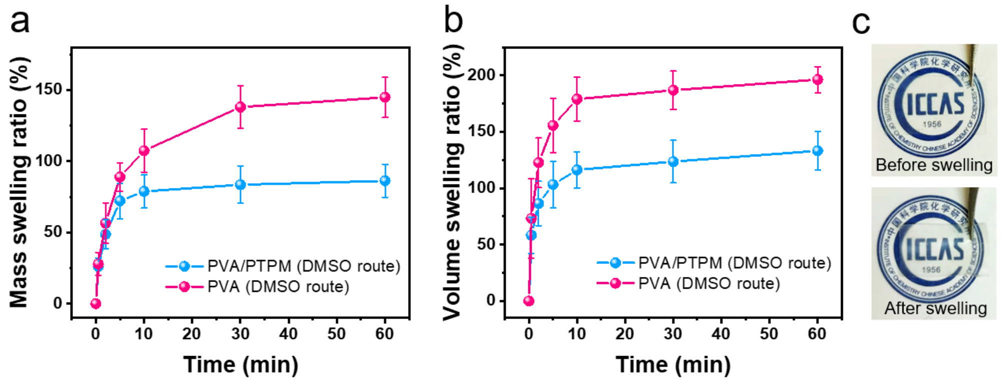

Figure S23. Mass (a) and volume (b) swelling ratio of PVA (DMSO route) and PVA/PTPM HN (DMSO route) in water over time. (c) Optical images of PVA/PTPM HN (DMSO route) before (top) and after (bottom) swelling in water.

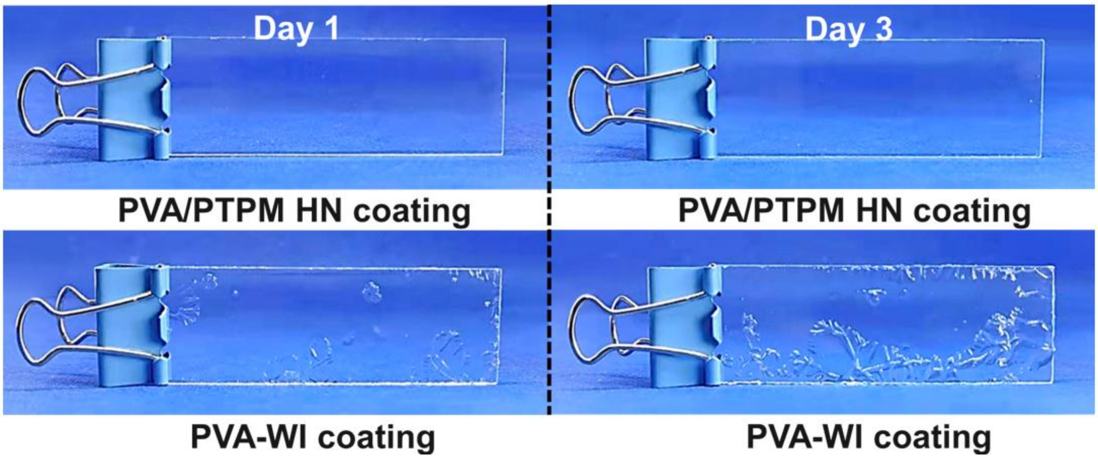

Figure S24. Optical images of PVA/PTPM HN (top) and PVA-WI (bottom) coatings after soaking in 20℃ water for 1 day and 3 days.

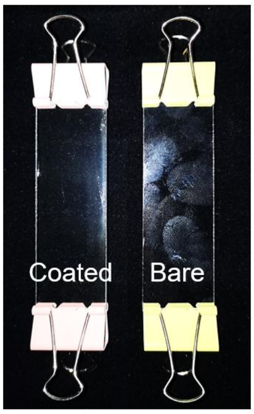

Figure S25. Fingerprints on PVA/PTPM HN coated (left) and uncoated (right) glass slides after rinsing with water.

References

[1] W. Zhang, X. Zou, F. Wei, H. Wang, G. Zhang, Y. Huang, Y. Zhang, *Compos. Pt. B-Eng*. **2019**, *162*, 500.

[2] X. Yu, J. Zhao, C. Wu, B. Li, C. Sun, S. Huang, X. Tian, *Mater. Des*. **2020**, *194*, 108956.
